# Assessment of Piglet Vitality by Farmers—Validation of A Scoring Scheme and Estimation of Associated Genetic Parameters

**DOI:** 10.3390/ani9060317

**Published:** 2019-06-04

**Authors:** Katharina Schodl, Regine Revermann, Christoph Winckler, Birgit Fuerst-Waltl, Christine Leeb, Alfons Willam, Peter Knapp, Christina Pfeiffer

**Affiliations:** 1 Division of Livestock Sciences, Department of Sustainable Agricultural Systems, University of Natural Resources and Life Sciences (BOKU), Vienna, Gregor-Mendel Strasse 33, 1180 Vienna, Austria; katharina.schodl@boku.ac.at (K.S.); regine.revermann@students.boku.ac.at (R.R.); christoph.winckler@boku.ac.at (C.W.); birgit.fuerst-waltl@boku.ac.at (B.F.-W.); christine.leeb@boku.ac.at (C.L.); alfons.willam@boku.ac.at (A.W.); 2 Schweinezuchtverband & Besamung OOE, 4641 Waldstraße 4, Austria; peter.knapp@szv.at

**Keywords:** pigs, piglet vitality, piglet mortality rate, litter size, genetic parameters, breeding program

## Abstract

**Simple Summary:**

The aim of animal breeding is to improve desirable traits in animals over generations by selecting those animals with the best performance for producing offspring. Sows have been bred for bearing and raising large litters. However, piglets in large litters are often underweight at birth and have a higher risk of dying before weaning. Therefore, breeding for large litters presents an animal welfare issue and focus should be put on new traits, such as piglet vitality. To select the best performing sows, breeders need a scheme to assess piglet vitality on a routine basis. In this study, 23 farmers used a four-point scoring scheme for piglet vitality (1 = low vitality to 4 = high vitality) to assess 3171 litters. To validate the new scoring scheme, i.e., to see if it assesses what it is supposed to assess, the vitality scores were compared to the piglet mortality rate of the respective litters. The results show that litters assessed with low vitality scores had the highest mortality rate and vice versa. Furthermore, genetic analysis showed that the trait is hereditary. Therefore, including piglet vitality into breeding programs may contribute to animal welfare improvement.

**Abstract:**

Maternal breeds for sows have been bred for high prolificacy during recent decades. Although large litters may be beneficial for economic efficiency, pre-weaning mortality is increased. Thus, focus should instead be put on new traits such as piglet vitality (PV). Until now, no validated scoring scheme for piglet vitality exists, which is feasible to be applied for routine on-farm trait recording. The objective of this study was to validate a four-point vitality scoring scheme (1 = low vitality to 4 = high vitality) applied by farmers based on pre-weaning mortality and to estimate genetic parameters. A linear mixed model was fitted for piglet vitality for 3172 litters from Large White and Landrace sows on 23 farms and correlations were calculated for vitality score and piglet mortality. A subsample of 2900 records was used for genetic analysis. Pre-weaning mortality differed significantly between all vitality score categories except for 1 and 2, ranging between 7.98% (category 4) and 29.1% (category 1). PV was genetically negatively correlated to litter size (−0.68) and mortality rate (−0.65), whereas litter size was positively correlated with mortality rate (0.59). Including PV into breeding programs may, thus, improve animal welfare.

## 1. Introduction

In recent decades, breeding goals for maternal breeds for sows have focused predominantly on increasing litter size. Although selection for this trait may be beneficial in terms of economic efficiency, (public) concern has been raised about negative side effects on animal health and welfare [1]. As a consequence, sustainable breeding goals should also include non-economic traits, such as animal-welfare-related traits [1,2]. Large litters are characterized by heterogeneous or generally low individual birth weights due to limited uterus capacity and inadequate nutrient supply [3]. Underweight piglets have a higher risk for chilling, starvation, and being crushed by the sow [4]. Consequently, selection for large litters increases pre-weaning mortality, and thus represents a serious animal welfare issue [5].

Furthermore, large litters require management measures during birth and lactation, such as split suckling, cross-fostering, or artificial rearing of piglets, which may also negatively affect piglet and sow welfare [6]. Besides animal welfare issues, these management measures require a higher input of labor and investment for equipment. While these may be easily available on larger farms with a high degree of specialization and mechanization, this may not be the case for small-scale family farms. Pig breeding in Austria is still in the hands of farmers, as opposed to the majority of European countries where animal breeding has been taken over by companies [7]. Many farms are run as family farms with various production branches. During workload peaks, such as harvest time, labor is a scarce resource, and thus cannot be dedicated to labor intensive management procedures for the pigs all year round. Furthermore, investment in expensive technologies may not be profitable for a small number of sows.

In light of these developments, Austrian pig breeding organizations and scientists are currently revising the breeding programs for maternal breeds with the aim to include maternal behavioral traits and to model novel breeding programs. With regard to litter quality, the focus was put on new traits to reduce pre-weaning mortality. Selection based on survival, vitality, and growth of piglets represents a good strategy for reducing piglet mortality [8].

Viability is the ability of a piglet to survive, whereas piglet vitality describes the strength and vigor of a piglet and also presents an important factor for survival [9,10]. Several studies (e.g., [10,11,12]) have addressed parameters to assess the vitality of piglets after birth, commonly referring to the so-called modified APGAR score introduced by Randall [13]. The original APGAR score is a clinical scoring system used in human medicine to describe the vitality of new born babies [14]. It uses five parameters: Appearance, Pulse, Grimace, Activity, and Respiration (APGAR). Revermann et al. [15] modified the APGAR score for piglets assessing skin color (normal (pink), pale, abnormal (blue)), respiration (within 15 s, after 15 s, irregular after 15 s), latency to first movement (much movement within 15 s, little movement within 15 s, no movement within 15 s), latency to first attempt to stand up (within one minute, after one to five minutes, after five minutes), latency to first teat contact (within ten minutes, 10–30 min, after 30 min), and condition of umbilical cord (connected, ruptured and minimum 15 cm long, ruptured and shorter than 15 cm). Due to the complex and time consuming data recording, the APGAR scoring system is not feasible for application on farms and more practical solutions to assess piglet vitality are needed. In field studies using piglet vitality as a female fertility trait, breeders classified litter vitality qualitatively using a point scale [16,17]. However, assessment schemes for the scores vary between these studies and validation is still missing. Based on the definition of vitality as the ability of a piglet to survive, pre-weaning mortality rate may be used to validate scoring schemes for piglet vitality [18].

Therefore, the aims of this study were: (1) to validate a litter vitality scoring scheme applied by farmers based on piglet mortality; and (2) to estimate heritabilities, genetic and phenotypic correlations for vitality score, pre-weaning mortality rate, and total number of born piglets to better understand the genetic background.

## 2. Animals, Materials and Methods

Altogether 23 farms in the three main pig producing counties in Austria (Styria, Lower, and Upper Austria) participated in the data collection between July 2017 and June 2018. Either Large White or Landrace sows were kept in farrowing crates, in compliance with Austrian animal welfare legislation, from five days before birth until the end of the suckling period [19], which lasted on average 28 days. Vitality was assessed at litter level within 24 h postpartum (starting from the expulsion of the placenta) by farmers according to a four-point scoring scheme (Table 1). Farmers were advised to categorize litters depending on the number of piglets showing signs of reduced vitality. These piglets appear weak and languid, their skin looks pale, and they show reduced activity and insufficient suckling. Usually, piglets have pink skin, move swiftly, and start suckling quickly after birth.

Farmers were trained twice to use the vitality scoring scheme appropriately. The first training was carried out as part of a workshop for a group of farmers, using photos as examples for each category. Immediately preceding the data collection, farmers were trained again, this time individually on-farm. Furthermore, farmers collected data on piglet mortality by recording the number of live-born piglets and the number of piglets that died during the suckling period, including also the cause of death. During the aforementioned training workshop for litter vitality scoring, farmers were also trained to distinguish between still-born piglets and live-born piglets who died immediately after birth. Furthermore, training included identification of the cause of death of piglets who died during the suckling period. Definitions for still-born piglets and different causes of death that were recorded by farmers are presented in Table 2. Data on vitality scoring and piglet mortality were recorded using a tablet with an adapted version of the “Sauenplaner” [20], a program used for routine performance data collection on breeding and multiplier farms.

After data were checked for plausibility, vitality scoring was analyzed for 2.493 litters from Large White sows and 679 litters from Landrace sows, with a maximum of three observations per sow. Piglet mortality was calculated according to Formula (1)

Mortality (%) = (number of dead piglets until weaning)/(live-born piglets) × 100(1)

whereby all litters with weaning dates later than 35 days after birth were discarded from the analysis.

Statistical analyses were performed using SAS 9.4 [21]. The level for significance was defined at *p* < 0.05. To describe the association between vitality score and piglet mortality a Spearman rank correlation (PROC CORR) was calculated. A linear mixed model (PROC MIXED) was fitted for piglet mortality [%] with vitality score (1 to 4), farm (1–23), obstetrics (yes, no), season (July–August, September–October, November–December, January–February, March–April, May–June), sow breed (Large White, Landrace), and parity (parity1, parity 2–3, parity 4–5, parity 6–7, and parity 8–13) as fixed effects, and sire (1–284) and sow (1970) nested within farm (23) as random and repeated effects, respectively. Tukey’s test was used to establish pair-wise differences between piglet mortality rates in the four vitality score categories. Furthermore, a logistic regression (PROC GLIMMIX) was performed to calculate odds ratios (odds ratio statement) and the probability (ilink statement) for piglet mortality for each vitality score category. Odds ratios were calculated for litters with and without dead piglets for each vitality score category. The final model included the binary outcome variable for piglet mortality (0 for litters with no dead piglets, 1 for litters with at least one dead piglet), farm (1–23), obstetrics (yes, no), season (defined as above), breed (Large White, Landrace), vitality score (1 to 4), and parity (parity1, parity 2–3, parity 4–5, parity 6–7 and parity 8–13) as fixed effects, and sire (1–284) and sow (1970) nested within farm (23) as random and repeated effects, respectively.

For the genetic analysis a subsample of 2900 records was used, including 2257 litters of Large White sows and 643 litters from Landrace sows from 22 farms. Additionally, farrowing classes were derived from farrowing age of the sow (in months) combined with parity, resulting in nine different farrowing classes in the dataset. The pedigree was traced back as far as possible, yielding 9772 animals. Heritabilities and genetics and phenotypic correlations were calculated fitting a trivariate linear animal model applying an average information algorithm using the ASReml package [22]. The vitality score is ordinally scaled; despite that, a linear model was fitted, as linear models are robust against deviations of the assumptions of normal distribution [23], and are thus usually used in routine genetic evaluations. The following model was applied:

y = Xb + Za + Wp + e(2)

where y indicates the vector of observations of the vitality score, pre-weaning mortality rate in percent (see formula above), and the number of total born piglets, respectively; b is the vector of fixed effects, comprising farm, breed of sow, season, as well as farrowing class; a is the vector of the random additive genetic effects of the sow; p is the vector of permanent environmental effects of the sow and e is the vector of the random residual effects. Vector a was assumed to have multivariate normal distribution (MVN), with MVN (0, G = G_0_ ⊗ A), where G_0_ is a 3 × 3 additive genetic variance-covariance matrix, ⊗ is the Kronecker product of matrices, and A represents the numerator relationship matrix. Vectors p and e represent the permanent environmental and random residual effects, with N (0, ${I\sigma }_{p}^{2}$) and N (0, I$\sigma_{e}^{2}$), where I is the identity matrix, $\sigma_{p}^{2}$ is the permanent environmental variance, and $\sigma_{e}^{2}$ is the residual variance. It was assumed that the permanent environmental and the residual covariance were zero. X, Z, and W represent the incidence matrices for fixed, random, and permanent environmental effects, respectively.

## 3. Results

Overall, the mean mortality rate was 12.6% with a standard deviation of 9.10%, and the mean vitality score was 3.64 ± 0.59. The correlation of r = −0.331 shows that piglet mortality was negatively correlated with vitality score. In other words, litters with a higher vitality score had a lower mortality rate.

### 3.1. Validation of the Litter Vitality Scoring System

Pre-weaning mortality was significantly affected by vitality score category, farm, season, and parity. More than half of all recorded litters were scored with vitality score 4 (69.7%). A quarter of all litters were rated with vitality score 3 (25.6%), 4.04% with vitality score 2, and only 0.69% with vitality score 1. Table 3 shows the mean piglet mortality rate for each vitality score category. Mortality rates for score categories 1 and 2 (low vitality scores) were equally high, whereas all others differed significantly, with the lowest mortality rate in score category 4 (high vitality score).

Table 4 shows the odds ratios for vitality scores 1 to 3 compared to vitality score 4, corresponding estimates, and 95% confidence intervals, as well as the probabilities for piglet mortality in each vitality score category. The 95% confidence intervals indicate that the vitality scores 1 to 3 all differed significantly from vitality score 4. Estimated probability for piglet mortality was highest for vitality score 2 (0.900).

### 3.2. Heritabilities, Genetic, and Phenotypic Correlations

The mean vitality score in the subsample for genetic parameter estimation was 3.66 ± 0.57. The mean number of total born piglets per sow was 14.10 ± 3.3 and the mean mortality rate was 12.8% ± 10.4%. Heritabilities and genetic and phenotypic correlations of all three traits are given in Table 5. All genetic parameters were significantly different from zero, and standard errors were relatively small.

## 4. Discussion

### 4.1. Method of Data Collection

In the present study, farmers were instructed to assess vitality within 24 h postpartum. Most of the pre-weaning mortality occurs 48 h after birth [24]. Hence, if the assessment was carried out towards the end of the 24 h, farmers may have assessed only the surviving piglets, which may have biased their judgement towards a higher vitality score. On the contrary, observing a high number of still-born piglets already during birth may have led to a lower vitality scoring by farmers. To produce more sound results, it would therefore be advisable to record the time elapsed after the end of farrowing with the vitality assessment and the number of piglets that had died between birth and time of assessment.

### 4.2. Validation of the Piglet Vitality Scoring System

LS-Means for pre-weaning mortality for each vitality score category showed that the number of dead piglets decreased from category 1 (low vitality) to category 4 (vital; Table 2). The absence of a significant difference between scores 1 and 2 may be due to the low number of litters that scored low in vitality (only 0.68% scored 1). The generalized regression model yielded similar results: the probability for mortality in a litter decreased with a higher vitality score (Table 3). The odds ratios showed that piglets in litters scored with vitality scores 1–3 had a higher chance to die than piglets in litters scored as category 4. The odds ratio for category 2 exceeded category 1, which may be due to the low number of litters scored 1. Another possible explanation is that litters scored 2 already displayed low vitality, and thus a differentiation between score 1 and 2 may not be decisive for predicting survivability of piglets in a litter. Following this approach, score 1 and 2 may be subsumed under one score for on-farm application.

The lower mortality rates found in the higher vitality score categories were also reflected in the rank correlation coefficient between vitality score and mortality rate, which indicated a medium strong correlation [25]. The correlation was negative, indicating that litters with a higher vitality score had a lower preweaning mortality rate. Other studies investigating the relationship between piglet vitality and mortality rate yielded similar results, even though most of the time an APGAR score was used for vitality assessment. De Roth and Downie [26], for example, found that piglets with a low score for clinical evaluation of vitality (comparable to the APGAR score) had a higher risk of dying during the first ten days of life. This relationship was also found by Trujilo-Ortega et al. [27], and to a lower extent also by Muns et al. [9], but not by Trujilo-Ortega et al. [28]. The medium strong correlation found in this study may be superimposed by the effect of different observers on the different farms. In the other studies mentioned above, vitality was assessed by only one observer.

Results show that although piglet survival is generally influenced by many different factors, the assessment scheme used in this study may serve as a valid scheme to assess piglet vitality. The low occurrence of score 1 and the similarly high mortality rates of litters rated with scores 1 and 2 suggest those two score categories should be subsumed, although this may entail a reduction of variance in the trait. Furthermore, efforts should be undertaken to reduce the effect of the different observers, which may be achieved by continuous training, for example using online tools or regular workshops. Implementing an additional inter-observer reliability testing may enable assessment of how the application of the assessment scheme by farmers develops. An online training tool may present a suitable method to combine training and inter-observer reliability testing [29].

### 4.3. Heritabilities and Genetic and Phenotypic Correlations

The heritability estimates found in this investigation were similar to those found in other studies. Gorssen et al. [30], for example, applied a four-scale scheme to assess Pietrain litters in Belgium for piglet vitality. Using a three trait sire-dam model, they estimated a heritability of 0.11. Klein et al. [15] also developed a four-scaled assessment scheme, which was applied by farmers on organic weaner production farms with German Landrace x German Large White sows. Using a linear animal model, they estimated a heritability of 0.14 ± 0.03. Lower heritabilities were reported by Stratz et al. [16]. In total, 90 farmers in Germany and Switzerland assessed piglet vitality for litters of purebred German Landrace, Large White, and Pietrain sows, as well as their crossings, using five categories. Genetic parameters were estimated using a linear mixed model and a threshold model, yielding heritabilities of 0.03 ± 0.01 and 0.07 ± 0.02, respectively. It has to be emphasized that the different studies found similar heritabilities for piglet vitality despite the use of different scales for piglet vitality (four-point scale vs. five-point scale), different number of assessors (one assessor vs. assessment by various farmers), and using different models to estimate genetic parameters (dam-sire models vs. animal models). Heritability for total number of piglets born was similar to those found by Lund et al. [31] in Landrace (0.11 ± 0.01) and Yorkshire (0.14 ± 0.02) sows, but higher than the heritability reported for sows in a nucleus population for maternal lines by Hermesch et al. [32] (0.06 ± 0.03). Heritability for mortality rate (0.09 ± 0.03), however, was similar to Hermesch et al. [32] (0.05 ± 0.03).

Klein et al. [15] estimated a moderate negative genetic correlation for piglet vitality and number of live-born piglets (r = −0.35 ± 0.16). In order to compare their results with the present study, the trivariate animal model was fitted again, using number of live-born piglets instead of total born piglets. The correlation of −0.59 ± 0.22 found in this study was higher than the correlation estimated by Klein et al. [15], indicating that breeding for a higher number of live-born piglets negatively affects piglet vitality. However, number of live-born piglets may not serve as a valid indicator when discussing the effect of breeding for large litters on piglet vitality. Due to the higher risk for still-birth associated with large litters [33], using number of live-born piglets may underestimate or produce incorrect information about the litter size. Fitting the model with total number of born piglets resulted in a stronger negative genetic correlation with lower standard errors (−0.68 ± 0.16), and thus underlines that the number of live-born piglets may present an inadequate indicator for litter size. Correlations between total number of born piglets and mortality rate were in line with other studies by Hermesch et al. [32] (0.76 ± 0.19) and Lund et al. [31], who reported a negative correlation of total number of born piglets with percentage of piglets alive after three weeks (−0.39 ± 0.07 and 0.01 ± 0.23 for Landrace and Yorkshire, respectively).

With regard to the heritability, the results show that genetic progress for piglet vitality can be achieved. The genetic correlations suggest that breeding for higher piglet vitality reduces the total number of born piglets but may also reduce preweaning mortality at the same time. Although litter size may be lowered, the number of weaned piglets may not be affected due to reduced mortality. Regarding the particular structure of pig breeding in Austria, breeding for more vital piglets and smaller litter size may be more suitable for family farms with limited resources in terms of manpower and investment in expensive technologies. This was confirmed by the positive feedback of the breeding organizations and breeders involved in this study [34]. However, further research on economic effects of smaller litters with more vital piglets should be conducted.

The results of this study suggest that including piglet vitality into breeding programs for maternal sow lines can contribute to pig welfare improvement. This may be achieved by routine trait recording by breeders using the proposed scoring scheme. Nevertheless, a combination of different litter quality traits, and thus a piglet vitality index is needed for future sustainable breeding programs.

## Figures and Tables

**Table 1:** Litter vitality scoring scheme used by farmers for assessment of vitality at litter level.

Vitality Score	Definition/Description
1	More than 4 piglets in the litter show signs of reduced vitality ^1^
2	3 to 4 piglets in the litter show signs of reduced vitality ^1^
3	1 to 2 piglets in the litter show signs of reduced vitality ^1^
4	No piglet shows signs of reduced vitality ^1^

^1^ Piglets appear weak, languid, pale, and show reduced activity and insufficient suckling.

**Table 2:** Causes of death recorded by the farmers.

Cause of Death	Definition/Description	Counted as
Still-born	Piglets that are born dead but are fully developed. They still have the “slippers” on their feet and are often mekonium stained	Still-born piglets
Crushed	Piglets found in the area where the sow lies down; often with twisted extremities and visible haematoma	Piglet mortality rate
Killed by sow	Piglets with injuries resulting from being bitten or stepped on by the sow (haematoma and wounds)	Piglet mortality rate
Starved	Piglets appearing emaciated with their ribs visible. Illnesses of the sow, such as recorderd mastitis-metritis-agalactia	Piglet mortality rate
Other causes	Piglets that died of other causes then the above	Piglet mortality rate

**Table 3:** Number of litters (*n*), Least Squares Means, and Standard Errors (SE) of piglet mortality rate [%] per vitality score category. Significant differences (*p* < 0.05, Tukey Test) between categories are indicated by letters a, b, c, d.

	Vitality Score			
	1	2	3	4
*n*	22	128	813	2.209
Piglet mortality rate (%)	29.12 ^a^	22.85 ^ab^	15.72 ^c^	7.98 ^d^
SE	2.64	1.26	0.76	0.67

**Table 4:** Odds ratios for piglet mortality in litters with vitality scores 1–3 compared to litters with vitality score 4 and probabilities [%] for piglet mortality per vitality score category. SE = Standard error.

Vitality Score	Reference Group	Odds Ratio	95% Confidence Interval		Probability for Mortality	SE
1	4	4.173	1.217	14.311	0.812	0.104
2	4	8.671	4.160	18.074	0.900	0.039
3	4	3.690	2.946	4.621	0.792	0.032
4	-	-	-	-	0.508	0.040

**Table 5:** Trivariately estimated heritabilities are shown on the diagonal and in bold type, genetic correlations on the upper triangle matrix, and phenotypic correlations on the lower triangle matrix.

	Vitality Score	Total Number of Piglets Born	Mortality Rate (%)
Vitaliy score	**0.11** ± **0.04**	−0.68 ± 0.16	−0.65 ± 0.18
Total number of piglets born	−0.28 ± 0.02	**0.19** ± **0.04**	0.59 ± 0.16
Mortality rate (%)	−0.33 ± 0.02	0.32 ± 0.02	**0.09** ± **0.03**

## References

[1] Kanis E., de Greef K.H., Hiemstra A., van Arendonk J.A.M. (2005). Breeding for societally important traits in pigs. J. Anim. Sci..

[2] Merks J.W.M., Mathur P.K., Knol E.F. (2012). New phenotypes for new breeding goals in pigs. Animal.

[3] Rutherford K.M.D., Baxter E.N., D’Eath R.B., Turner S.P., Arnott G., Roehe R., Ask B., Sandøe P., Moustsen V.A., Thorup F. (2013). The welfare implications of large litter size in the domestic pig I: Biological factors. Anim. Welf..

[4] Edwards S.A. (2002). Perinatal mortality in the pig: Environmental or physiological solutions?. Livest. Prod. Sci..

[5] Prunier A., Heinonen M., Quesnel H. (2010). High physiological demands in intensively raised pigs: Impact on health and welfare. Animal.

[6] Baxter E.M., Rutherford K.M.D., D’Eath R.B., Arnott G., Turner S.P., Sandøe P., Moustsen V.A., Thorup F., Edwards S.A., Lawrence A.B. (2013). The welfare of large litter size in the domestic pig II: Management factors. Anim. Welf..

[7] Preisinger R. (2004). Internationale Tendenzen der Tierzüchtung und die Rolle der Zuchtunternehmen. Züchtungskunde.

[8] Leenhouwers J.I., de Almeida Júnior C.A., Knol E.F., van der Lende T. (2001). Progress of farrowing and early postnatal pig behavior in relation to genetic merit for pig survival. J. Anim. Sci..

[9] Baxter E.M., Jarvis S., D’Eath R.B., Ross D.W., Robson S.K., Farish M., Nevison I.M., Lawrence A.B., Edwards S.A. (2008). Investigating the behavioural and physiological indicators of neonatal survival in pigs. Theriogenology.

[10] Mota-Rojas D., Nava-Ocampo A.A., Trujillo M.E., Velázquez-Armenta Y., Ramírez-Necoechea R., Martinez-Burnes J., Alonso-Spilsbury M. (2005). Dose minimization study of oxytocin in early labor in sows: Uterine activity and fetal outcome. Reprod. Toxicol..

[11] Mota-Rojas D., Trujillo M.E., Martínez J., Rosales A.M., Orozco H., Ramírez R., Sumano H., Alonso-Spilsbury M. (2006). Comparative routes of oxytocin administration in crated farrowing sows and its effects on fetal and postnatal asphyxia. Anim. Reprod. Sci..

[12] Randall G.C. (1971). The relationship of arterial blood pH and pCO_2_ to the viability of the newborn piglet. Can. J. Comp. Med..

[13] Apgar V. (1966). The Newborn (Apgar) Scoring System: Reflections and Advice. Pediatr. Clin. N. Am..

[14] Revermann R., Winckler C., Fuerst-Waltl B., Leeb C., Pfeiffer C. (2018). Assessment of viability of new born piglets using an adjusted APGAR score. JCEA.

[15] Klein S., Brandt H.R., König S. (2018). Genetic parameters and selection strategies for female fertility and litter quality traits in organic weaner production systems with closed breeding systems. Livest. Sci..

[16] Stratz P., Just A., Faber H., Bennewitz J. (2016). Genetic analyses of mothering ability in sows using field-recorded observations. Livest. Sci..

[17] Elbert K., Tetens J., Wassmuth R. (2018). Vitale Ferkel—Der Einfluss der Wurfgröße—Eine Übersichtsarbeit. Züchtungskunde.

[18] BMGF Verordnung der Bundesministerin für Gesundheit und Frauen über die Mindestanforderungen für die Haltung von Pferden und Pferdeartigen, Schweinen, Rindern, Schafen, Ziegen, Schalenwild, Lamas, Kaninchen, Hausgeflügel, Straußen und Nutzfischen (1. Tierhaltungsverordnung). https://www.ris.bka.gv.at/GeltendeFassung.wxe?Abfrage=Bundesnormen&Gesetzesnummer=20003820.

[19] Intelicon Software Development GmbH, SPonWeb. https://www.intelicon.eu/smallpigweb.

[20] SAS Institute Inc. (2013). Base SAS® 9.4 Procedures Guide: Statistical Procedures.

[21] Gilmour A.R., Gogel B.J., Cullis B.R., Thompson R. (2009). ASReml User Guide Release 3.0..

[22] Koeck A., Egger-Danner C., Fuerst C., Obritzhauser W., Fuerst-Waltl B. (2010). Genetic analysis of reproductive disorders and their relationship to fertility and milk yield in Austrian Fleckvieh dual-purpose cows. J. Dairy Sci..

[23] Marchant J.N., Rudd A.R., Mendl M.T., Broom D.M., Meredith M.J., Corning S., Simmers P.-H. (2000). The timing and causes of piglet mortality due to crushing on an open farrowing system. Vet. Rec..

[24] Cohen J. (1988). Statistical Power Analysis for the Behavioural Sciences.

[25] De Roth L., Downie H.G. (1976). Evaluation of viability of neonatal swine. Can. Vet. J..

[26] Trujillo-Ortega M.E., Mota-Rojas D., Juárez O., Villanueva-García D., Roldan-Santiago P., Becerril-Herrera M., Hernández_González R., Mora-Medina P., Alonso-Spilsbury M., Rosales A.M. (2011). Porcine neonates failing vitality score: Physio-metabolic profile and latency to the first teat contact. Czech J. Anim. Sci..

[27] Muns R., Manzanilla E.G., Sol C., Manteca X., Gasa J. (2013). Piglet beahviour as a measure of vitality and its influence on piglet survival and growth during lactation. J. Anim. Sci..

[28] Trujillo-Ortega M.E., Mota-Rojas D., Olmos-Hernández A., Alonso-Spilsbury M., González M., Orozco H., Ramírez-Necoechea R., Nava-Ocampo A.A. (2007). A study of piglets born by spontaneous parturition under uncontrolled conditions: Could this be a naturalistic model for the study of intrapartum asphyxia?. Acta Biomed..

[29] Schenkenfelder J., Winckler C. (2017). Development and evaluation of an online training tool for the assessment of animal based welfare parameters in cattle. ACS.

[30] Gorssen W., Jannsens S., Buysn N. The value of commercial farm-management data to evaluate Pietrain boars for vitality and robustness. Proceedings of the 69th Annual Meeting of the European Federation of Animal Science.

[31] Lund M.S., Puonti M., Rydhmer L., Jensen J. (2002). Relationship between litter size and perinatal and pre-weaning survival in pigs. Anim. Sci..

[32] Hermesch S., Luxford B.G., Graser H.-U. (2001). Genetic parameters for piglet mortality, within litter variation of birth weight, litter size and litter birth weight. Proc. Assoc. Advmt. Anim. Breed. Genet..

[33] Vanderhaeghe C., Dewulf J., de Kruif A., Maes D. (2013). Non-infectious factors associated with stillbirth in pigs: A review. Anim. Reprod. Sci..

[34] Pfeiffer C.P., Schodl K., Fuerst-Waltl B., Willam A., Leeb C., Winckler C. (2018). Developing an optimized breeding goal for Austrian maternal pig breeds using a participatory approach. JCEA.

